# Accuracy of Computer-Assisted Dynamic Navigation as a Function of Different Intraoral Reference Systems: An In Vitro Study

**DOI:** 10.3390/ijerph18063244

**Published:** 2021-03-21

**Authors:** Sigmar Schnutenhaus, Anne Knipper, Martin Wetzel, Cornelia Edelmann, Ralph Luthardt

**Affiliations:** 1 Centre for Dentistry, Dr Schnutenhaus Community Health Centre (CHC) GmbH, 78247 Hilzingen, Germany; knipper@schnutenhaus.de (A.K.); wetzel@Schnutenhaus.de (M.W.); edelmann@Schnutenhaus.de (C.E.); 2 Department for Dentistry, Clinic for Prosthodontics, Ulm University, 89081 Ulm, Germany; ralph.luthardt@uniklinik-ulm.de

**Keywords:** surgery, computer-assisted, computer-aided surgery, dental implants, computer-guided surgery, dynamic navigation, real-time tracking

## Abstract

The aim of this in vitro study was to determine whether the process chain influences the accuracy of a computer-assisted dynamic navigation procedure. Four different data integration workflows using cone-beam computed tomography (CBCT), conventional impressions, and intraoral digitization with and without reference markers were analyzed. Digital implant planning was conducted using data from the CBCT scans and 3D data of the oral models. The restoration of the free end of the lower jaw was simulated. Fifteen models were each implanted with two new teeth for each process chain. The models were then scanned with scan bodies screwed onto the implants. The deviations between the planned and achieved implant positions were determined. The evaluation of all 120 implants resulted in a mean angular deviation of 2.88 ± 2.03°. The mean 3D deviation at the implant shoulder was 1.53 ± 0.70 mm. No significant differences were found between the implant regions. In contrast, the workflow showed significant differences in various parameters. The position of the reference marker affected the accuracy of the implant position. The in vitro examination showed that precise implantation is possible with the dynamic navigation system used in this study. The results are of the same order of magnitude that can be achieved using static navigation methods. Clinical studies are yet to confirm the results of this study.

## 1. Introduction

The objective of prosthetic implant restoration is to restore the masticatory organs after tooth loss. Therefore, the functional and esthetic rehabilitation should be as natural as possible [1]. The long-term success of implant restoration is determined by multiple factors [2]. When planning implant positions, various aspects must be considered and assessed equally. For example, the bone condition [3], soft tissue condition [4], distances between the implants and neighboring teeth [5], and position of the cement space [6] must be considered while planning an implant position. Prosthetic-driven planning is shown to be suitable for achieving this goal in an optimal and predictable manner [7]. A previous study reported implants placed using computer-assisted procedures had a similar one-year survival rate as implants placed via a conventional procedure [8]. However, patient pain and discomfort are significantly reduced when a flapless procedure is used [9]

Digital 3D planning has been used to achieve favorable implant positioning. The actual condition of the alveolar bone is recorded using three-dimensional imaging (computed tomography (CT) or cone beam computed tomography (CBCT)), and merged with the target of a digitized prosthetic planning goal [1]. Computer-assisted procedures enable the implementation of digital implant planning [10]. In static navigation, drill templates are used to implement the planning. The use of drill templates for implants has been extensively examined [11]. This procedure has proven to be clinically accurate and achieves predictable results [12,13]. Various studies regarding the accuracy of static navigation have identified influencing factors including intraoral positioning and fixation of templates [14]. The manufacturing process for the drill template can also impact the accuracy of implant placement, as can the materials used [15,16,17]. Several studies have reported the influence of different drill sleeves on accuracy [18,19,20]. However, major inaccuracies in the implementation can often be traced back to application errors, not to the actual process [21].

In addition to static computer-assisted surgical procedures, dynamic procedures are also available [1]. The preparation of the implant bed and implant insertion are achieved by a surgeon who navigates the oral cavity with a three-dimensional representation of the actual implant bed on a screen [22]. The positions of the instruments are recognized in real-time using optical tracking systems with defined reference markers and are displayed on a screen [23]. These procedures have been introduced in various preclinical studies [24,25,26]. The development of computer technology and associated computer-aided methods has increased the use of dynamic navigation in clinical practice in recent years [1]. Due to the open-source systems, any implant systems can be used with dynamic navigation; however, it is not possible to store a static template using these systems [1]. The implantation is conducted with real-time visualization, allowing for intraoperative modifications of the plan [13]. Dynamic navigation can also be used when there is little vertical space, unlike drill templates [27]. However, the complexity of the surgical procedure requires sufficient training, and a learning curve has been reported [28]. The results of a systematic review on the accuracy of dynamic computer-assisted navigation revealed comparable clinical outcomes to those of static navigation. However, there is heterogeneity among individual dynamic navigation systems that must be considered [29]. The development of surgical implant procedures based on virtual and augmented reality technologies has resulted in an increase in the quality of restoration [30].

The aims of this in vitro study were to determine influencing factors on the accuracy of a dynamic navigation procedure and to clarify whether the workflow of implant planning and the implementation of 3D planning affect accuracy. In addition, we examined whether the position of the implant and the resulting relative position of the marker affect the accuracy.

## 2. Materials and Methods

In this controlled in vitro study, four process chains (Table 1) of data integration for implementing dynamic navigation were examined. In addition, the accuracy of the overall system and variables related to the implant region were analyzed. Fifteen identical models made of hard plastic (mandible B6 Bone Standard, GOS Göttinger OP-Simulationssysteme, Northeim, Germany) were used for each of the four process chains. These models represented a partially edentulous lower jaw with a unilateral free-end in regions 45–48 that required restoration.

### 2.1. Implantation Planning and Models

Individual planning for the two implants was performed for each model using implant planning software (coDiagnostiX Version 9.11, Dental Wings GmbH, Chemnitz, Germany). The planning data were loaded and digitally assigned to each other.. The planning data differed for each process chain.

An implant in region 45 (bone level tapered, BLT) with a diameter of 3.3 mm and length of 10 mm (Straumann Institut AG, Basel, Switzerland) and an implant in region 47 (BLT) with a diameter of 4.1 mm and length of 10 mm (Straumann Institut AG, Basel, Switzerland) were planned. The implant planning was based on prosthetic principles. The implants were centered on the ridge in a vestibular-lingual alignment. The distance between the two implants was 15 mm in each case. The implants were aligned parallel to one another using the parallelization function of the program. After the planning was completed, the data were transferred to the navigation system. The CBCT, planning, and marker positioning data were converted into a form that the navigation system could read using a function of the coDiagnostiX planning software. All planning steps were performed by an experienced dentist (A.K.).

**Figure 1:**
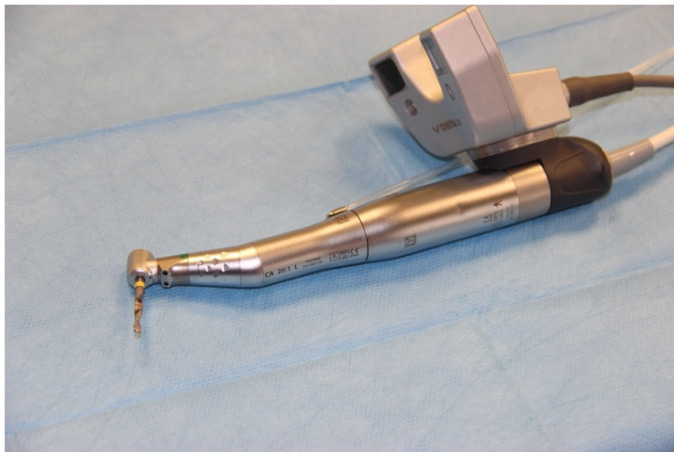
Surgical handpiece with camera attached.

**Figure 2:**
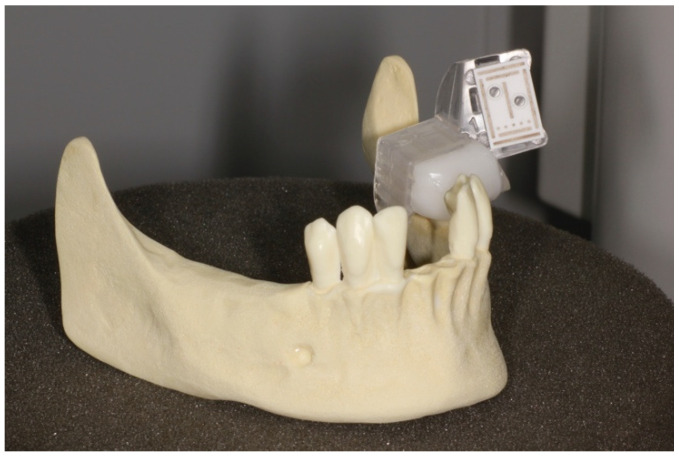
Plastic model with fixed marker, in this example a model from Group A.

The data were transferred to a DENACAM navigation system (Mininavident AG, Liestal, Switzerland). The DENACAM system works with a camera attached to the surgical handpiece (Figure 1). A marker is placed in the mouth, or in this case, on the model as a reference structure (Figure 2). The system is guided using a 3D display on a screen that is clearly visible to the surgeon. The position, angle, and depth are displayed in real-time.

**Figure 3:**
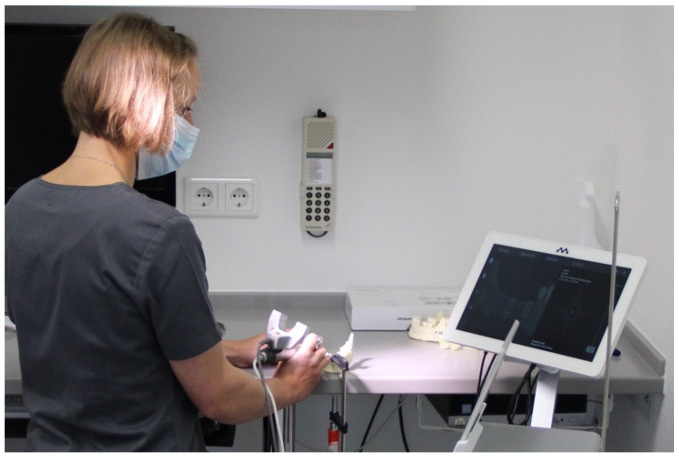
Implementing implantation under standardized laboratory conditions.

The models were fixed in a stable position on a worktop according to the prescribed protocol (Figure 3). Implantation was performed according to the drilling protocol provided by the implant manufacturer. All model implantations were performed by an experienced dentist (A.K.) using a contra-angle handpiece. The end position of the implant was determined using the DENACAM system’s display. Neither visual control nor readjustment of implant position was performed.

**Figure 4:**
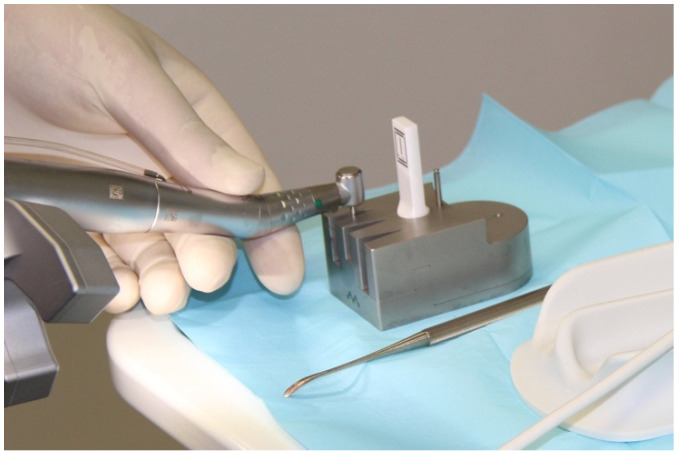
Registering the drill.

Each drill, including the implant, was automatically registered with a registration instrument before its use (Figure 4). Each drill was inserted in the surgical contra-angle handpiece and placed in a registration block with the marker for automatic registration. The dimensions of the drill were measured. This measurement was compared to the manufacturer’s specifications. This procedure was performed each time the drill was changed to provide a system with precise information on the length and diameter of the drill being used.

### 2.2. Process Chains

CBCT with a marker and intraoral digitization without a marker (A): a prefabricated holder of the reference marker (DENATRAY, Mininavident, Liestal, Switzerland) was attached to the model with a synthetic thermoplastic material (DENABEADS, Mininavident, Liestal, Switzerland). It was positioned counter-laterally to the implantation region within regions 36 and 37. The marker, a plate made of zirconium oxide ceramic with defined characteristics, was inserted into the tray. The model was positioned with the marker placed on a holder in a digital volume tomograph (CBCT, Gendex CB500, Gendex Dental Systems, Des Plaines, IL, USA). The CBCTs were performed with a standardized resolution of 0.2 voxels. After CBCT was performed, the tray holder was removed and intraoral digitization of the model was performed using a Trios 3 scanner (3Shape A/S, Copenhagen, Denmark). For implant planning, CBCT and intraoral digitization of the model were read into the planning software. During model implantation, the holder with the marker was repositioned in the same place on the model teeth.

CBCT without a marker and intraoral digitization with and without a marker (B_1): a CBCT was produced from the model without the use of a reference marker using the settings described in process A. Intraoral digitization of the jaw was carried out. Once intraoral digitization was complete, a holder with the marker was placed in the same manner described in process A. A subsequent intraoral digitization was created with the holder and marker in place. For implant planning, the CBCT image and intraoral digitization were input into the planning software without a marker and with a marker. During model implantation, the holder and marker were in place.

CBCT without a marker and conventional impression and extraoral digitization with and without a marker (B_2): this approach is a variant of process B_1 that uses the more conventional approach of an alginate impression of the jaw (Blueprint cremix, Dentsply DeTrey, Constance, Germany) instead of intraoral digitization. A model was constructed from a super hard stone in the dental laboratory. The model was then scanned in a laboratory scanner (E4, 3Shape A/S, Copenhagen, Denmark) with and without an attached marker. The CBCT image and placement of the marker were performed as described above. For implant planning, the CBCT image and extraoral digitization of the plaster model were input into the planning software with and without markers. During model implantation, the holder and marker were in place.

CBCT without a marker and intraoral digitization without a marker (C): a CBCT image and intraoral digitization of the jaw were each performed without a reference marker. The resulting datasets were used for implant planning. After the planning was complete, a 3D object representing a holder for the reference marker was inserted into the planning. The object was placed in the contralateral position to the implant region, in regions 34–36, above the teeth. A reference marker holder that could be securely attached to the teeth in quadrant III was made using the drill templates. The design data of the marker templates were sent to an in-house dental laboratory. All marker templates were created by a dental technician using a 3D printer (Version 3, Formlabs Inc., Somerville, MA, USA). The templates were cleaned and post-cured according to the manufacturer’s instructions. During model implantation, the printed holder and marker were in place.

### 2.3. Registering the Implant Position

After implantation, the scan bodies were screwed onto the two implants. The models were then optically digitized using a high-precision laboratory scanner (E4, 3Shape A/S, Copenhagen, Denmark) and a surface dataset was generated and saved as a standard tessellation language (STL) file. These datasets were integrated into the original digital plans for the evaluation.

**Figure 5:**
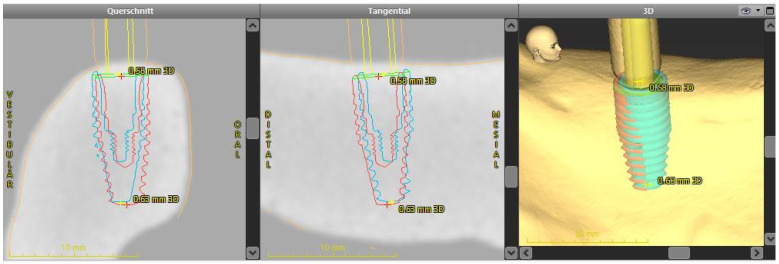
Overlay of the planned and actual achieved implant position using the treatment evaluation program function of coDiagnostiX software.

The automated surface best-fit matching using the iterative closest point algorithm in the treatment evaluation mode of the coDiagnostix software was used to overlay the preoperative CBCT with the postoperative lab scans (Figure 5). The overlay and evaluation were performed by a dentist (M.W.) who was not involved in the planning or implant placement.

### 2.4. Analysis of the Implant Position

The metric analysis included the following measurements:

- 3D deviation: the three-dimensional deviation of the midpoints between implant planning and the clinically-achieved implant position, measured at the implant shoulder and apex (corresponding to the Euclidean distance).
- Apico-coronal deviation (height difference): vertical spatial offset measured at the center of the implant shoulder.
- Axis deviation: Angular deviation of the implant axes between the planned and clinically-achieved implant positions.
- The two-dimensional deviations in the mesio-distal and bucco-lingual directions were measured at the implant shoulder and at the implant axis.

### 2.5. Statistical Analysis

Variables are described as means with standard deviations, 95% confidence intervals (CIs), and minimum and maximum values. The Shapiro–Wilk test was used to determine the normality of the distribution of the data. The groups were compared using the analysis of variance or Kruskal–Wallis tests as appropriate. Tukey’s post-hoc test was performed for normally-distributed data, and the Mann–Whitney U-test was used to compare data without normal distribution. Post-hoc tests were used to identify significant differences between the groups.

All statistical analyses were conducted using SPSS^®^ Statistics version 27 (IBM Corp. Released 2020, Armonk, NY, USA). Statistical significance was set at *p* < 0.05.

## 3. Results

A total of 120 implants in 60 oral models were evaluated. The coronal 3D deviation was significantly different between the different process chains (*p* < 0.05).

The mean 3D deviation at the implant shoulder of all 120 implantations was 1.53 mm (95% CI: 1.40–1.66 mm). The mean angular deviation was 2.88° (95% CI: 2.51–3.25°). The data for all measured values are shown in Table 2.

There were no significant differences in the positional deviations between implants in region 45 and implants in region 47 (Table 2).

The accuracy was different between the four process chains (Table 3). The workflow had no effect on the angular deviation (F (3, 116) = 1.003; *p* = 0.394), coronal mesiodistal deviation (F (3, 116) = 0.386; *p* = 0.763), apical mesiodistal deviation (F (3, 116) = 0.701; *p* = 0.553), coronal horizontal deviation (F (3, 116) = 1.068; *p* = 0.365), or apical horizontal deviation (F (3, 116) = 1.228; *p =* 0.303).

Overall, process chain B_1 resulted in the greatest deviations (Table 4). The mean 3D deviation at the implant shoulder resulting from process chain B_1 (1.85 ± 0.52 mm) was significantly greater than that achieved by process chains A (1.40 ± 0.65 mm), B_2 (1.48 ± 0.92 mm), and C (1.39 ± 0.59 mm). The mean bucco-lingual deviation measured at the shoulder resulting from process chain B_1 (1.47 ± 0.65 mm) was also significantly greater than that achieved by process chains A (0.60 ± 0.48 mm), B_2 (0.91 ± 0.75 mm), and C (0.95 ± 0.53 mm).

**Figure 6:**
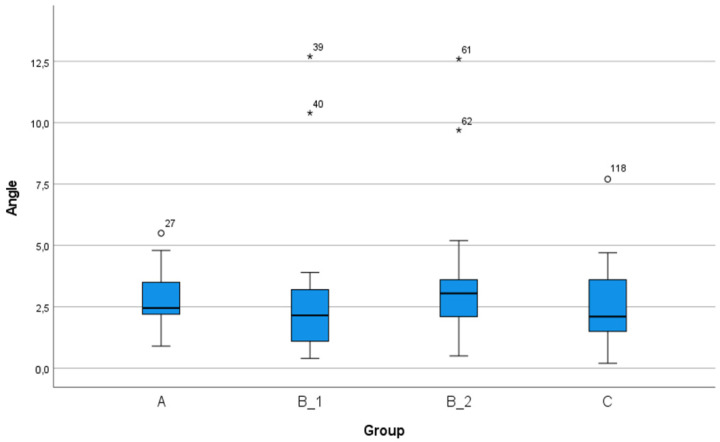
The angular deviations achieved by each process chain. No significant differences were observed between the process chains. Process chain B_1: CBCT without a reference marker and intraoral digitization with and without a reference marker. Process chain B_2: CBCT without a reference marker and conventional impression and extraoral digitization with and without a reference marker. Process chain C: CBCT without a reference marker and intraoral digitization without a reference marker.

**Figure 7:**
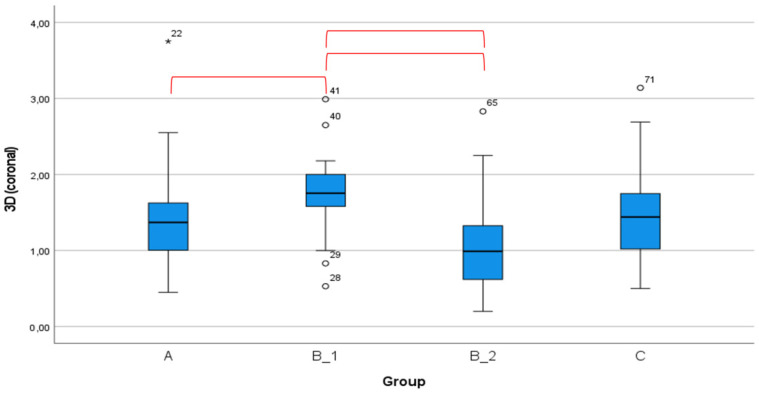
The global 3D deviations at the coronal exit point achieved by each process chain. The 3D deviations at the coronal exit point are significantly different between the B-1 process chain and the A, B_2, and C process chains. Statistical significance was determined using the Mann–Whitney U test. The red lines indicate significant group differences.

There were no significant differences in angular deviation between any of the four process chains (Figure 6). The global 3D deviation measured at the coronal end of the implant was significantly different between process chain B_1 and process chains A, B_2, and C, as shown in Figure 7.

## 4. Discussion

No significant differences were found between the implant regions. In contrast, the workflow showed significant differences in various parameters. The position of the reference marker affected the accuracy of the implant position. The in vitro examination showed that precise implantation is possible with the dynamic navigation system used in this study.

To date, few in vitro studies have examined the accuracy of implant positions achieved using dynamic navigation. The first reports were published in 2005 [24,26,31], with subsequent publications nearly 10 years later [32,33,34,35,36,37]. Different tracking systems were examined in each of these studies. The reference markers were partially distributed at a large distance from the patient and later were included in the patient’s field of vision. In this study, the markers were placed intraorally to reduce the complexity of the structure and to achieve a setting that is adapted to the requirements of oral surgery.

The mean angular deviation in the present study was 2.88 ± 2.03°. These values show a high level of precision compared to angular deviations reported in previous studies that ranged from 1.09 ± 0.55° [37] to 12.37 ± 4.18° [32]. The unweighted mean value from the studies mentioned above is 4.4°. In this study, the angular deviation was not affected by the process chain or the implant region.

The deviation at the implant exit point has prosthetic importance as inclined implant axes make designing the proximal contacts difficult in cases where individual abutments are not used. In particular, the exit point of the implant directly affects the esthetic results [38]. The mean value of the global 3D deviation at the coronal end of the implant in this study was 1.53 ± 0.70 mm, which is more accurate than the previously-reported values of 0.41 ± 0.12 mm to 1.58 ± 0.80 mm [29]. In this study, the mean mesio-distal deviation was 0.70 ± 0.59 mm and the mean bucco-lingual deviation was 0.98 ± 0.68 mm, which are consistent with the previously reported results of lineal deviations of 0.33 ± 0.19 mm to 3.03 ± 1.81 mm [29]. In this study, the height offset of the 3D-deviation is significant. The final horizontal position was achieved using the navigation system display, and subsequent corrections were not made. The height of the implant in relation to the crestal bone can typically be verified and corrected intraoperatively if the implant planning includes a sufficient safety distance in the axial direction [39].

The subgroup analysis of the implant regions revealed that the distance between the implant and the marker had no significant influence on the accuracy between the planned and clinically-achieved implant positions. The bucco-lingual deviations varied the most between process chains. In this study, the bucco-lingual deviation was affected by the differences in the process chains.

Various factors that influence the accuracy of static navigation have been reported including template design, template positioning and fixation, and surgical access (open vs. closed) [40]. However, the type of implant can affect the accuracy of implantation [41]. In this study, a conical bone-level implant was used in each implantation. Therefore, it is not clear whether the accuracy of implant placement is influenced by the macrodesign of the implant or the drill sequence. These factors may be clinically relevant. To avoid possible uncontrolled co-factors, only one implant system was used in this study. However, the results of an in vitro study cannot be used to directly support clinical use. For example, factors such as bone density, bone anatomy, residual teeth, mouth opening, and patient movements may also affect the accuracy of the implant position and are not accounted for in in vitro studies. In addition, the placement of the marker is better controlled on models than with patients. In this study, only one standard mandible position, the unilateral free-end position of the mandible, was investigated. The intraoral fixation of the reference marker in cases of significantly reduced residual dentition or complete edentulism has not yet been described.

Mediavilla-Guzmán et al. examined the differences in accuracy between a static and dynamic approach in an in vitro study and reported that only the angular deviation was significantly different between the two approaches (static approach: 2.95 ± 1.48°; dynamic approach: 4.00 ± 1.41°) [35]. In another in vitro study that compared dynamic and static methods, each accuracy parameter that was measured was found to be significantly different, with the dynamic system achieving less accuracy; however, the deviation values were much higher than those found in this study [32].

The experience of the surgeon has a minor influence on the accuracy achieved with a static approach [42]. However, the experience of the surgeon has been reported as an influencing factor when dynamic navigation is used [36]. In this study, the experience of the surgeon was found to play a minor role in the accuracy of the implantation. Implant osteotomy can be performed under optimized conditions. Clinical studies are necessary to investigate the effect of the experience and skill of the surgeon on accuracy and the learning curve. Therefore, when comparing systems, the difficulty of using the system in clinical practice should be examined in addition to the accuracy of the system.

The transferability of in vitro results to clinical practice has yet to be proven for the dynamic system presented here. In a systematic review of static navigation, significant differences were found between in vitro and clinical studies, including differences in the apical horizontal deviation and the angular deviation [43]. Similar results were reported in a meta-analysis by Schneider et al. [44]. Factors affecting the transferability of these results to a clinical setting include the patient’s ability to open the mouth, patient movements, or a restricted view of the operating field [45].

In contrast to static navigation, relatively few clinical studies regarding dynamic navigation have been published to date [46,47,48,49,50,51,52]. These studies have reported heterogeneous factors affecting accuracy. Different dynamic navigation systems, implant planning programs, and implants were used in these previous studies, resulting in mean angular deviations between 2.26 ± 1.62° and 6.46 ± 3.95°. The previously reported 3D deviations at the coronal end of the implant were between 0.67 ± 0.29 mm and 1.37 ± 0.55 mm.

Previous studies have reported similar average deviation values obtained with dynamic and static navigation systems [11,43,45]. However, the accuracy varies greatly in these clinical and in vitro studies. More studies are required to determine whether this is due to the specific dynamic navigation system. In addition, some systems have only been evaluated by one surgical team. The accuracy and feasibility of these systems for clinical use must be examined in future studies. To assess the benefits of this procedure for the patient, the operation time, surgical difficulty, and cost must also be evaluated.

## 5. Conclusions

The in vitro study showed that a sufficiently precise implantation is possible with the dynamic navigation system used in this study. The workflow influenced the implant placement accuracy. Accuracy is dependent on the navigation system being used. Clinical studies are needed to verify the results of this study and to assess the clinical feasibility of dynamic navigation.

## Figures and Tables

**Table 1:** Overview of the four different process chains.

		Workflow			
		A	B_1	B_2	C
Data Generation	CBCT	CBCT image with marker	CBCT image	CBCT image	CBCT image
	On the patient	Intraoral scan	Two intraoral scans (with and without marker)	Alginate impression	Intraoral scan
	Virtually				Creation of a digital marker template
	In the laboratory			Two model scans (with and without marker)	3D printing of a marker template
Reference marker in surgery		Surgery with Denatray	Surgery with Denatray	Surgery with Denatray	Surgery with marker template

**Table 2:** Deviations between the planned and clinically-achieved implant positions.

	Total *n* = 60 Models/120 Implants			Region 45 *n* = 60 Models/60 Implants			Region 47 *n* = 60 Models/60 Implants			*p*-Value
	Mean (SD)	95% CI	Min–Max	Mean (SD)	95% CI	Min–Max	Mean (SD)	95% CI	Min–Max	
Deviation at implant shoulder (mm)										
3D	1.53 (0.70)	1.40–1.66	0.20–4.02	1.52 (0.64)	1.36–1.69	0.26–4.02	1.54 (0.77)	1.34–1.74	0.20–3.75	0.923
Mesio-distal	0.70 (0.59)	0.59–0.80	0.02–3.06	0.63 (0.57)	0.48–0.78	0.02–2.37	0.77 (0.62)	0.61–0.93	0.04–3.06	0.208
Bucco-lingual	0.98 (0.68)	0.86–1.11	0.00–2.81	1.09 (0.66)	0.91–1.25	0.00–2.52	0.87 (0.70)	0.71–1.07	0.00–2.81	0.116
Apico-coronal	0.57 (0.50)	0.48–0.67	0.00–2.34	0.48 (0.43)	0.37–0.60)	0.00–2.17	0.66 (0.54)	0.51–0.80	0.00–2.34	0.059
Deviation at implant apex (mm)										
3D	1.79 (0.80)	1.64–1.94	0.29–4.05	1.81 (0.74)	1.78–2.00	0.29–4.05	1.77 (0.86)	1.54–1.99	0.29–3.74	0.766
Mesio-distal	0.81 (0.70)	0.68–0.93	0.01–3.73	0.80 (0.71)	0.62–0.98	0.04–3.73	0.82 (0.69)	0.64–1.00	0.01–3.26	0.890
Bucco-lingual	1.25 (0.75)	1.12–1.39	0.01–3.10	1.36 (0.66)	1.19–1.53	0.20–2.79	1.15 (0.83)	0.93–1.36	0.01–3.10	0.126
Apico-coronal	0.58 (0.50)	0.49–0.67	0.00–2.35	0.50 (0.43)	0.39–0.61	0.00–2.19	0.66 (0.55)	0.52–0.81	0.00–2.35	0.068
Angular deviation (°)	2.88 (2.03)	2.51–3.25	0.20–12.70	2.87 (2.22)	2.30–3.44	0.20–12.70	2.89 (1.83)	2.41–3.36	0.40–10.40	0.964

**Table 3:** Deviations between the planned and clinically-achieved implant positions.

	Process Chain A *n* = 15 Models/30 Implants			Process Chain B_1 *n* = 15 Models/30 Implants			Process Chain B_2 *n* = 15 Models/30 Implants			Process Chain C *n* = 15 Models/30 Implants			*p*-Value
	Mean (SD)	95% CI	Min–Max	Mean (SD)	95% CI	Min–Max	Mean (SD)	95% CI	Min–Max	Mean (SD)	95% CI	Min–Max	
Deviation at implant shoulder (mm)													
3D	1.40 (0.65)	1.16–1.64	0.41–3.75	1.85 (0.52)	1.66–2.04	0.53–2.99	1.48 (0.92)	1.14–1.83	0.20–4.02	1.39 (0.59)	1.17–1.61	0.50–3.14	0.034
Mesio-distal	0.80 (0.63)	0.55–1.04	0.06–3.06	0.64 (0.50)	0.45–0.83	0.04–1.70	0.68 (0.66)	0.44–0.93	0.02–2.37	0.67 (0.56)	0.46–0.88	0.04–2.63	0.763
Bucco-lingual	0.60 (0.48)	0.42–0.78	0.06–1.91	1.47 (0.65)	1.47–1.72	0.14–2.6	0.91 (0.75)	0.63–1.19	0.00–2.81	0.95 (0.53)	0.76–1.15	0.00–1.81	<0.005
Apico-coronal	0.67 (0.53)	0.47–0.87	0.00–2.10	0.56 (0.38)	0.41–0.70)	0.01–1.29	0.60 (0.65)	0.36–0.84	0.01–2.34	0.45 (0.37)	0.31–0.59	0.02–1.65	0.303
Deviation at implant apex (mm)													
3D	1.54 (0.72)	1.27–1.81	0.75–3.56	2.13 (0.62)	1.90–2.09	0.69–3.49	1.80 (1.06)	1.41–2.20	0.29–4.05	1.68 (0.65)	1.44–1.92	0.63–3.68	0.029
Mesio-distal	0.80 (0.66)	0.55–1.05	0.04–2.87	0.67 (0.51)	0.48–0.86	0.04–1.80	0.83 (0.88)	0.50–1.16	0.04–3.73	0.93 (0.70)	0.66–1.19	0.01–3.26	0.553
Bucco-lingual	0.86 (0.59)	0.64–1.08	0.01–2.41	1.82 (0.68)	1.57–2.07	0.08–3.10	1.23 (0.79)	0.93–1.20	0.12–2.93	1.10 (0.60)	0.88–1.32	0.18–2.31	<0.005
Apico-coronal	0.69 (0.53)	0.49–0.89	0.00–2.12	0.57 (0.39)	0.42–0.71	0.01–1.35	0.62 (0.65)	0.38–0.86	0.01–2.35	0.45 (0.37)	0.31–0.59	0.00–1.62	0.303
Angular deviation (°)	2.77 (1.06)	2.37–3.16	0.90–5.50	2.70 (2.61)	1.73–3.68	0.40–12.70	3.43 (2.44)	2.52–3.13	0.50–12.6	2.62 (1.60)	2.02–3.22	0.20–7.70	0.394

*p*-values were determined using the analysis of variance (ANOVA) test. Process chain A: cone-beam computed tomography (CBCT) with a reference marker and intraoral digitization without a reference marker. Process chain B_1: CBCT without a reference marker and intraoral digitization with and without a reference marker. Process chain B_2: CBCT without a reference marker and conventional impression and extraoral digitization with and without a reference marker. Process chain C: CBCT without a reference marker and intraoral digitization without a reference marker.

**Table 4:** Comparison of the deviations achieved by each process chain.

	A-B_1	A-B_2	A-C	B_1-B_2	B_1-C	B_2-C
Deviation at implant shoulder (mm)						
3D	0.001 *	0.722	0.873	0.003 *	0.002 *	0.844
Mesio-distal	0.747	0.887	0.849	0.992	0.997	1.000
Bucco-lingual	<0.001 *	0.220	0.125	0.003 *	0.007 *	0.992
Apico-coronal	0.800	0.937	0.303	0.988	0.835	0.648
Deviation at implant apex (mm)						
3D	0.022 *	0.573	0.905	0.366	0.119	0.929
Mesio-distal	0.884	0.998	0.897	0.805	0.481	0.951
Bucco-lingual	<0.001 *	0.147	0.502	0.005 *	<0.001 *	0.881
Apico-coronal	0.796	0.958	0.255	0.976	0.787	0.536
Angular deviation (degree)	0.999	0.590	0.992	0.513	0.998	0.413

The *p*-values for the comparisons are shown. The values were compared using Tukey’s test, with the exception of 3D deviations, which were compared using the Mann–Whitney U test. * indicates statistical significance.

## Data Availability

Not applicable.
